# Novel venous balloon for compliance measurement and stent sizing in a post-thrombotic swine model

**DOI:** 10.3389/fbioe.2023.1298621

**Published:** 2023-11-22

**Authors:** Mengjun Wang, Xiao Lu, Ling Han, Amy M. Wang, Seshadri Raju, Ghassan S. Kassab

**Affiliations:** ^1^ 3DT Holdings LLC, San Diego, CA, United States; ^2^ California Medical Innovations Institute, San Diego, CA, United States; ^3^ The Rane Center at St. Dominic’s Hospital, Jackson, MS, United States

**Keywords:** post-thrombotic stenosis, large animal model, stent sizing, compliance, vein

## Abstract

**Objective:** Real-time accurate venous lesion characterization is needed during endovenous interventions for stent deployment. The goal of this study is to validate a novel device for venoplasty sizing and compliance measurements.

**Methods:** A compliance measuring sizing balloon (CMSB) uses real-time electrical conductance measurements based on Ohm’s Law to measure the venous size and compliance in conjunction with pressure measurement. The sizing accuracy and repeatability of the CMSB system were performed with phantoms on the bench and in a swine model with an induced post thrombotic (PT) stenosis in the common femoral vein of swine.

**Results:** The accuracy and repeatability of the CMSB system were validated with phantom bench studies of known dimensions in the range of venous diameters. In 9 swine (6 experimental and 3 control animals), the luminal cross-sectional areas (CSA) increased heterogeneously along the PT stenosis when the CMSB system was inflated by stepwise pressures. The PT stenosis showed lower compliance compared to the non-PT vein segments (5 mm^2^ vs. 10 mm^2^ and 13 mm^2^ at a pressure change of 40 cm H_2_O). Compliance had no statistical difference between venous hypertension (VHT) and Control. Compliance at PT stenosis, however, was significantly smaller than that at Control and VHT (*p* < 0.05, ANOVA).

**Conclusion:** The CMSB system provides accurate, repeatable, real-time measurements of CSA and compliance for assessment of venous lesions to guide interventions. These findings provide the impetus for future first-in-human studies.

## Introduction

Chronic venous disease (CVD) is much more prevalent and affects a younger population than arterial disease. It is estimated that CVD affects 10%–35% of adults in the USA at treatment costs of $1–3B annually. A similarly high prevalence has been reported in the European populations, with incidence increasing with age (Maurins, et al., 2008; Robertson, et al., 2013). There have been major advances in diagnosing, classifying, and treating CVD in the last 4 decades. New and simpler techniques to treat superficial venous disease (saphenous reflux) have evolved. Because of the low morbidity and excellent results, saphenous reflux is typically addressed first before treating deep venous disease. The focus on deep venous disease has shifted from reflux (a traditional concept) to obstruction, which appears more prevalent than reflux (Labropoulos et al., 1997; Neglen et al., 2003). Obstruction can be corrected by minimally invasive endovenous stenting with low morbidity, excellent patency, and good clinical outcome.

Neglen and associates reported the results of iliac vein stenting to treat chronic venous disease in a large series of 982 limbs in a landmark paper published in 2008. They reported secondary patencies of 100% in 518 non-PT limbs and 86% in 464 PT limbs at 6 years (Neglen et al., 2007). In a recent analysis of 545 limbs that underwent iliac vein stenting, Jayaraj and others reported significant improvement in limb pain and swelling; 73% of venous ulcerNeglen et al., 2007). In a recent analysis of 545 limbs that underwent iliac vein stenting, Jayaraj and others reported significant improvement in limb pain and swelling; 73% of venous ulcers healed. Quality of life (Chronic Venous Insufficiency QOL Questionnaire, CIVIQ) score improved from 60 to 36 (*p* < 0.0001) at a median follow-up of 26 months. There was no mortality, and morbidity was minimal (Jayaraj et al., 2021a). Similar good patency and clinical results have been reported worldwide (Raju, 2013; Razavi et al., 2022). Because of significantly reduced morbidity and minimally invasive technique stent treatment in CVD has supplanted open deep venous surgery rendering it obsolete in clinical practice (Raju, 2015; Satwah et al., 2022). This is a significant shift in the chronic venous disease treatment paradigm that focused on the reflux component over the last two centuries. Interestingly, associated reflux does not worsen after iliac vein stenting, instead, it improves over time (Raju et al., 2022). Residual reflux does not appear to impair clinical outcome after stent treatment (Raju et al., 2010; Raju et al., 2013; de Graaf et al., 2015; Saleem et al., 2023).

Many aspects of iliac vein stenting are evolving, and ‘best practice’ standards remain unsettled while the experience matures. Some areas needing improvement are already obvious. There is a great need for a reliable diagnostic standard for detecting and grading iliac vein stenosis (Jayaraj et al., 2021b). Traditional techniques such as contrast venography, MR venography, and duplex ultrasound are wanting in diagnostic accuracy (Gagne et al., 2017; Raju et al., 2021; Saleem et al., 2022). Intravascular ultrasound (IVUS) has emerged as a *de facto* standard for diagnosis and grading of stenosis severity (Gagne et al., 2018; Montminy et al., 2019). IVUS may not be able to adequately image about 15%–25% of lesions located near confluences (Montminy et al., 2019; Raju et al., 2021). This ‘missing border’ deficit is due to the lack of a centering mechanism in available IVUS probes. CT venography has much fewer technical limitations of this sort that reduces applicability in patients (Raju et al., 2020). CT venography, however, is unsuitable for procedural guidance in most settings.

Here, we introduce a novel diagnostic balloon catheter that utilizes a law of physics (Ohm’s law) to determine the size and compliance of the vein objectively, in real-time. The technology is validated in phantoms and in a swine model of stenosis. The novel sizing/compliance measuring sizing balloon (CMSB) system showed excellent performance in both phantoms and *in vivo* peripheral vein with post thrombotic (PT) stenosis. The clinical utility and limitations of the technology are considered.

## Materials and methods

**FIGURE 1:**
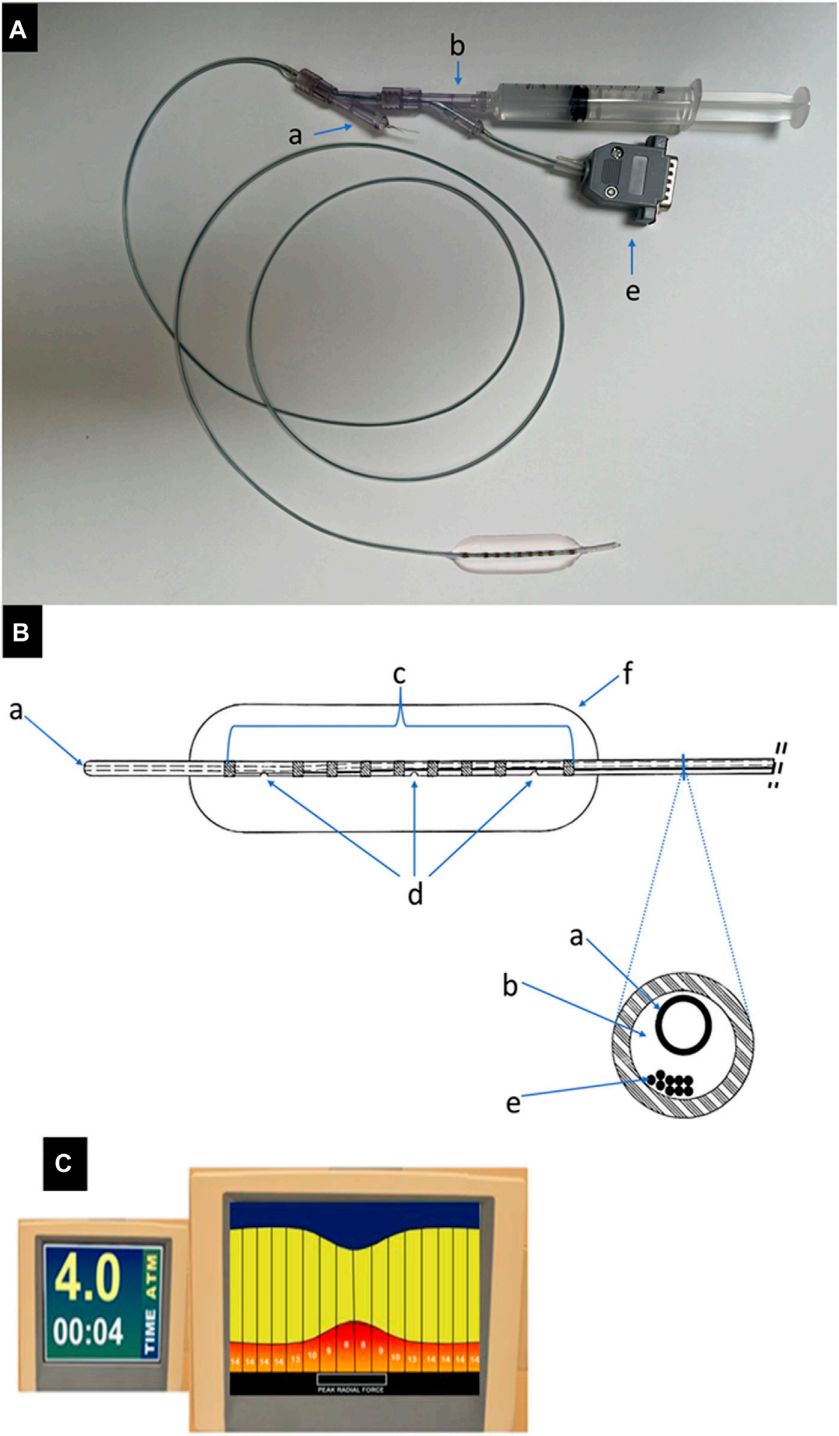
The CMSB system. **(A)**. An example of the CMSB system (catheter diameter 5 Fr, length 140 cm and balloon diameter 15 mm) with the guidewire channel (a), filling and pressure channel (b) and impedance connector cable (e). **(B)**. The line drawing of the distal balloon part of the CMSB system. (a): guidewire channel; (b): filling and pressure channel; (c): copper electrodes, the device has multiple electrode pairs along the length of the balloon to provide a complete profile over the 8 measurements; (d): filling and pressure ports, (e): electrode wires (impedance connector cable), (f): built-in flexible balloon. **(C)**. The console is a monitor and data acquisition system that 1) Generates the current across the outer electrodes, 2) Measures voltage drops across the inner electrodes, 3) Calculates the balloon sizes based on Ohm‘s Law, and 4) Displays the sizing profile data in real-time on the screen.

The compliance measuring sizing balloon (CMSB) system consists of three components: 1) CMSB system, 2) Impedance connector cable, and 3) Console. The CMSB system functions as a standard venoplasty balloon containing a 5 Fr catheter (140 cm in length) and a flexible balloon (2–18 mm in diameter and 60 mm in length). An inner guidewire channel and a channel for balloon inflation and pressure monitoring are integrated in the 5 Fr catheter (Figures 1A, B). The catheter segment in the balloon is covered by seven electrodes in the central region and two radiopaque outer marker electrodes at each end of the balloon. The outer marking electrodes also serve additional purposes for the CMSB system by working as detection electrodes and stimulating a constant electrical current between them. The electric current stimulated within the balloon is an alternating 136 μA, 10 kHz waveform, which has been shown to be safe when applied directly within the vasculature and within an insulated balloon eliminating the effects of parallel conductance (Isner et al., 1991; Kassab et al., 2004; Kassab et al., 2005; Kassab et al., 2009; Hermiller et al., 2011; Svendsen et al., 2014). During balloon inflation and deflation, detection electrodes make balloon sizing and solution conductivity measurements; the two outermost electrodes are positioned 10 mm away from the neighbor detection electrodes while the remaining seven detection electrodes are spaced at 5 mm intervals to enable 8 measurements in total (Figure 1B). A novel flexible circuit design provides multiple measurements made inside the balloon profile. It can be used to locate the minimum diameter during inflation (the venous lesion) as well as the reference diameter of the vessel away from the lesion. Additional functionality of the CMSB system is provided by integrating the balloon pressure into the console to display pressure and compliance. The console is a monitor and data acquisition system that 1) Generates the current across the outer electrodes, 2) Measures voltage drops across the inner electrodes, 3) Calculates the balloon sizes based on Ohm’s Law, and 4) Displays the sizing profile data in real-time on the screen using a 10 kHz sample rate on a continuously recording multiplex acquisition platform (Figure 1C).

### Bench Validation

The bench tests of the CMSB system were performed to verify the insulation of the balloon membrane and impedance cable. Further, the accuracy and repeatability of the CMSB system were performed on the bench using rigid phantoms, which were described in detail in our prior studies (Kassab et al., 2004; Kassab et al., 2005; Kassab et al., 2009; Hermiller et al., 2011; Svendsen et al., 2014; Svendsen et al., 2015). The plastic phantoms ranged from 2 to 18 mm in diameter and were independently measured to determine the true dimension. The value for the electrical conductivity of the injected 0.9% saline in the balloon was determined using an independent set of phantoms, and then randomized repeat measurements were made. The recorded voltage values and the other known parameters were used to calculate the measurements of the CMSB system.

The accuracy of the CMSB system was determined and validated by comparison of the difference between the CMSB system measurements and the true phantom sizes (considered the gold standard). Repeatability was determined and validated as the difference between consecutive randomized repeat CMSB system measurements in the same phantom.

### In-vivo animal validation

The CMSB system was tested in the femoral veins of nine Yorkshire swine (weight 40–60 kg). Six swine were assigned to the experimental group (tricuspid regurgitation and right femoral vein injury), and three swine were assigned to the control group. Preparation of the PT animals was a two-step process to activate all three elements of Virchow’s triad (Albadawi et al., 2017). Venous hypertension and reflux flow (activates the endothelium) in the femoral veins were induced by the initial creation of tricuspid reflux. As previously described, this was achieved by the avulsion of one or more chordae with a percutaneous avulsion device inserted through the right internal jugular vein in anesthetized animals (Brass et al., 2015; Lu et al., 2022). The degree of regurgitation induced was controlled by progressive avulsion of the chordae under intra-cardiac echography and doppler reflux-flow measurements. The target level of reflux was achieved when the atrial pressure was nearly equal (≤2 mmHg) to the ventricular pressure. This controlled level of tricuspid reflux produced venous hypertension without overt heart failure. The animals were allowed to recover and were monitored for 2 to 3 weeks before a second-step procedure.

The second process was to induce right femoral vein thrombosis while the left femoral vein was used as a control. The animals were anesthetized again, and the right femoral vein was traumatized by a balloon (Equalizer™ 7 Fr. 27 mm × 65 cm, Boston Scientific, Marlborough, MA, USA) introduced percutaneously through the right jugular vein. The inflated balloon was dragged across the venous lumen several times, causing endothelial injury. Venous flow in the traumatized vein was balloon-occluded at the outflow end of the traumatized segment for 2 hours thereafter. A small amount of thrombin (D-StatTM Flowable Hemostat Thrombin, 5000 US Units) was diluted into 10 mL of buffered water, Vascular Solution, Inc., Minneapolis, MN, USA) was injected into the traumatized segment to initiate thrombosis. After 7 weeks, PT stenosis evolved in the right femoral vein and no stenosis was observed in the left femoral vein.

The test of the CMSB system was evaluated in the right femoral vein with PT stenosis and the untreated left femoral vein (control) without stenosis in the experimental group. The test of the CMSB system was also evaluated in both right and left femoral veins in control animals where traumatic thrombosis was not induced. The CMSB system was positioned percutaneously in the upper right femoral vein via a long introducer sheath through the right jugular vein. Venography was performed with contrast media to determine the exact location of the testing site and the PT stenosis. The CMSB system was advanced over the stenosis, and the catheter was calibrated. In all left femoral veins in the experimental and control groups and right femoral veins in the control group, the CMSB system was positioned in all control segments without stenosis to mirror its location relative to the segment length in the experimental group with PT stenosis. The CMSB system was pressurized by raising the water level in a connected container. The pressure vs. CMSB size relationship was recorded at rest and with progressive intra-balloon pressures from 0 to 40 cm H_2_O at 5 cm H_2_O pressure intervals. At the end of the studies, animals were euthanized. The femoral veins were harvested to perform histological examinations.

### Statistical analysis

The average, standard deviation (SD), and root mean square (RMS) error were calculated for the accuracy and repeatability measurements. A linear regression (forced through the origin in line with Ohm’s law) and Bland-Altman analyses were performed for the accuracy (CMSB vs. true) and repeatability (CMSB measurement #1 vs. #2) data as well (Matin Bland and Altman, 1986). The venous compliance (CSA versus pressure) was measured at three states. The comparison of circumferential stretch (CSA) ratios among the three states was analyzed using Analysis of Variance (ANOVA), with (*) p-valve <0.05 indicating a significant difference.

### Ethical animal care

All animal experiments were performed at the California Medical Innovations Institute in accordance with national and local ethical guidelines, including the Institute of Laboratory Animal Research guidelines, Public Health Service policy, Animal Welfare Act, and an approved California Medical Innovations Institute IACUC protocol regarding the use of animals in research.

## Results

**FIGURE 2:**
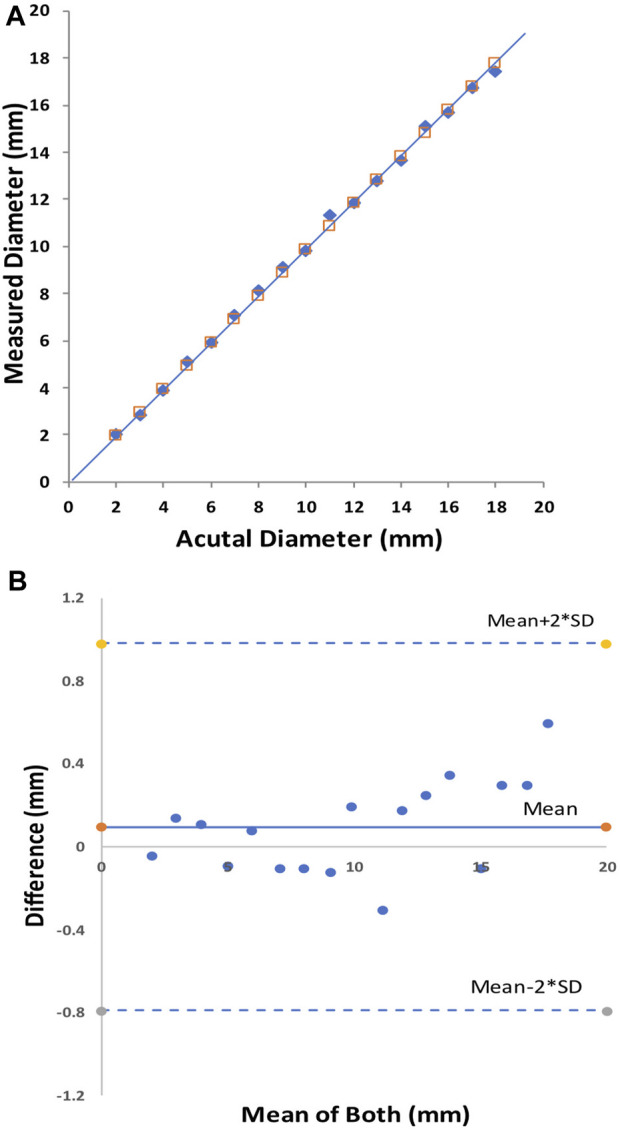
The CMSB bench accuracy in phantoms from 2 to 18 mm. Plot shows the identity relationship between the CMSB measured diameter and the actual diameter (solid black line as the identity line) **(A)** and the Bland-Altman analysis **(B)**.

**FIGURE 3:**
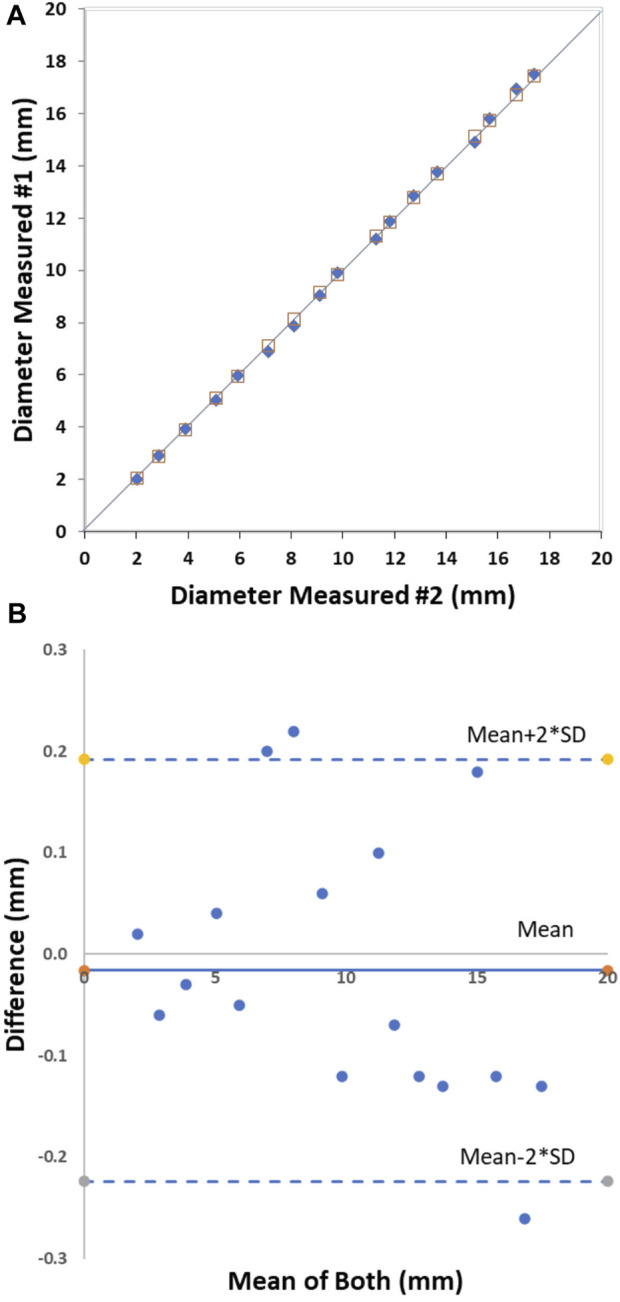
The CMSB bench repeatability in phantoms from 2 to 18 mm. Plot shows the identity relationship between the CMSB measured diameter and the actual diameter (solid black line as the identity line) **(A)** and the Bland-Altman analysis **(B)**.

The initial bench tests verified that the CMSB system met the expectation of the design in the study. The accuracy and repeatability were well within acceptable limits, consistent with the data from our multiple prior studies (Kassab et al., 2004; Kassab et al., 2005; Kassab et al., 2009; Hermiller et al., 2011; Svendsen et al., 2014; Svendsen et al., 2015). The accuracy of the CMSB system measurement was −0.10 ± 0.23 mm with an RMS error of 2.2% as compared to the actual phantom diameter on the bench (Figures 2A, B). The relationship between the CMSB measured diameter (D_measured_) and the actual diameter (D_actual_) was D_measured_ = 0.99*D_actual_; *R* ^2^ = 1.0 (Figure 2A **)**. For the repeatability analysis, the mean difference between repeat bench measurements was 0.02 ± 0.13 mm with an RMS error of 1.4% (Figures 3A, B). The repeatability relationship was D_measured_ #2 = 1.0*D_measured_ #1; *R* ^2^ = 1.0 (Figure 3A **)**.

**FIGURE 4:**
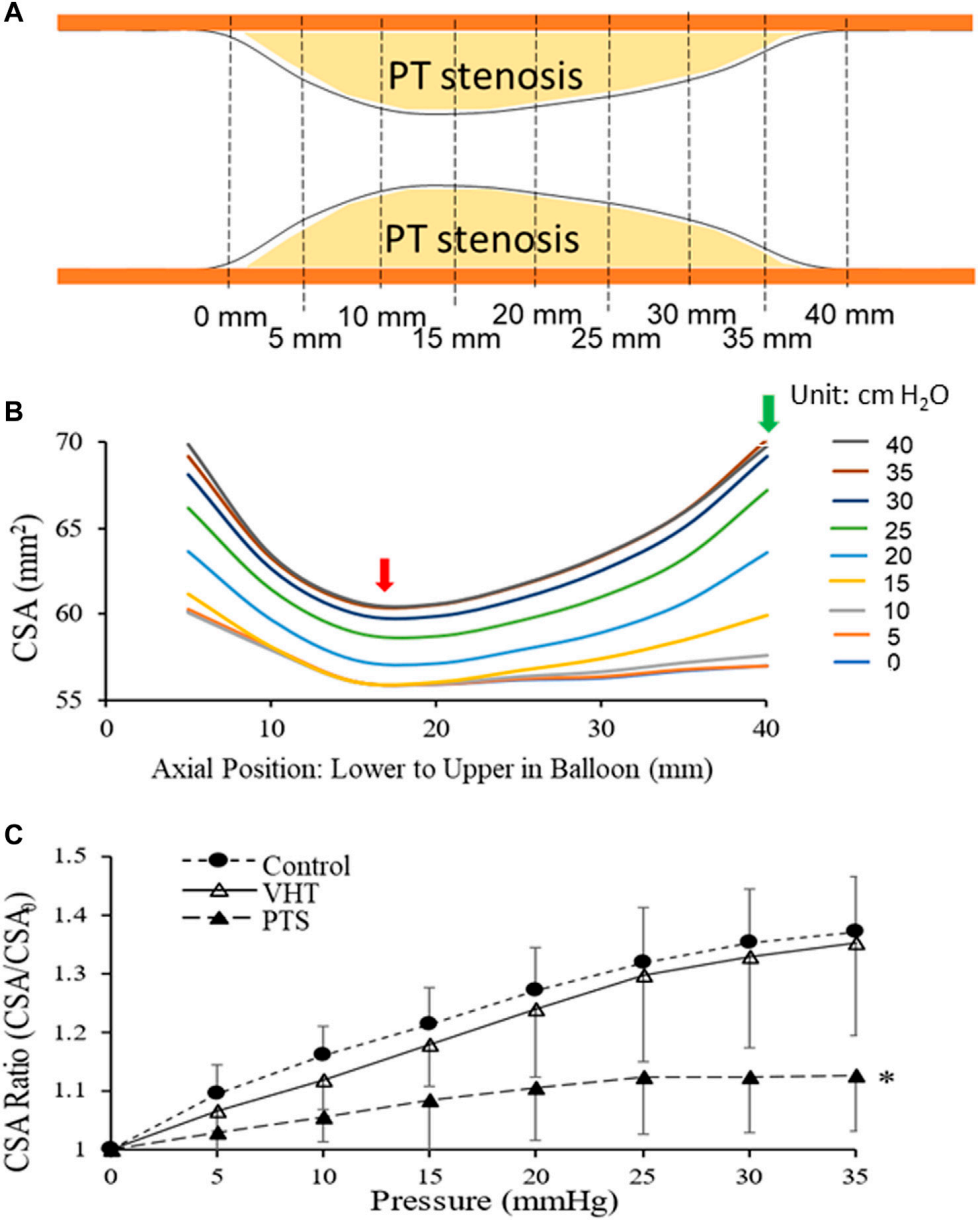
A balloon inflated by stepwise pressures changed the luminal CSA of a PT stenosis. **(A)** Illustration of PT stenosis formed in the femoral vein in the animal model. Both ends of the venous segment are venous lumen without stenosis. **(B)** An example of luminal CSA increased heterogeneously along the stenosis when a balloon catheter was placed at the stenosis and inflated by stepwise pressures. The CSAs at different balloon positions delineate the stenosis’s contour. The venous CSA and the filling pressure showed a characteristic non-linear relationship. It implicates heterogeneous stiffnesses of the venous segment at different positions. The site of the largest PT stenosis was the least compliant (red arrow), and the non-PT portion of the vein proximal to the PT stenosis was the most compliant (green arrow). **(C)** Venous compliance (the circumferential stretch ratio versus pressures relationship) at three states: control (Control), venous hypertension-induced tricuspid injury (VHT), and PT stenosis (PTS). Here the circumferential stretch ratios (CSA/CSA_0_) were the average of the ratios over the balloon length. *: *p* < 0.05 (ANOVA) in comparison with Control or VHT.

There were no arrhythmias, adverse events, or complications during CMSB system usage. The luminal CSA increased heterogeneously along the PT stenosis when the balloon was inflated by stepwise pressures. The contour of the stenosis was delineated with CSAs at different positions along the balloon (Figures 4A, B). The CSAs in the non-PT vein segments at the two ends were 60 and 57 mm^2^ at a recorded balloon pressure of 0 and 5 cm H_2_O, which increased to 70 mm^2^ at a balloon pressure of 40 cm H_2_O, which was the highest recorded pressure in the vein. The vein caliber had narrowed to 57 mm^2^ where the PT changes were maximum. The CSAs increased with the given balloon pressures at this location and reached 61 mm^2^ at 35 and 40 cm H_2_O. The venous CSA and the filling pressure showed a characteristic non-linear relation which suggests heterogeneous compliances (stiffnesses) of the venous segment at various axial positions. The site of the largest PT stenosis is the least compliant. The CMSB system revealed the morphometric and mechanical heterogeneities of PT stenosis.

Figure 4C displays venous compliance (the circumferential stretch ratio versus pressures relationship) at three states: Control (Control), venous hypertension-induced tricuspid injury (VHT), and PT stenosis (PTS). Here the circumferential stretch ratios (CSA/CSA_0_) were the average of the ratios over the balloon length. At both Control and VHT, there was an approximately linear increase in stretch ratio with increasing pressure (0–35 cm H_2_O). At PTS, before the pressure increased to 20 cm H_2_O (0–20 cm H_2_O), the stretch ratio increased linearly, and when the pressure reached and exceeded 25 cm H_2_O (25–35 cm H_2_O), the stretch ratio no longer increased with the pressure, entering a stretch ratio plateau, indicating that the compliance reached a limit in the pressure range. The venous compliance of VHT was lower than that of Control, but there was no statistical difference between them. The compliance at PTS, however, was significantly less than those at Control and VHT (*p* < 0.05, ANOVA).

## Discussion

The performance of CMSB system on the bench and *in vivo* shows safe, accurate, and repeatable sizing results, along with a demonstration of the utility for real-time pressure-diameter and CSA measurements. The CMSB system revealed the morphometric and mechanical heterogeneities of PT stenosis. Our study results validates that this novel CMSB system can accomplish the following: 1) Function as a compliant venoplasty balloon; 2) Provide CSA profile measurements (i.e., multiple measurements along the length of the vein); and 3) Utilize CSA balloon surface area (SA) and pressure to establish a profile of venous compliance (ΔCSA/ΔP) to guide stent selection and delivery in real-time in the swine model.

CMSB system is accomplished using accurate, physics-based electrical conductance measurements on a standard 5 Fr venoplasty balloon catheter. We have shown in other applications that electrical conductance measurements are a safe and highly effective method for assessing luminal organ dimensions (Kassab et al., 2004; Kassab et al., 2005; Kassab et al., 2009; Hermiller et al., 2011; Svendsen et al., 2014; Svendsen et al., 2015). The vein size can be determined accurately in real-time by stimulating a small alternating electrical current within the balloon. Within the balloon are multiple sets of measurement electrodes that provide an axial sizing profile along the length of the balloon. Multiple measurements along the length of the balloon provide a benefit because it does not require the physician to precisely place the middle of the balloon in the lesion at inflation, which is often difficult to achieve. The CMSB system is also innovative in incorporating a pressure measurement during inflation/deflation to determine regions within the balloon and vessel segment length with higher or lower compliance. This second feature of the CMSB system is key as it allows the physician to identify regions of the vessel that may not be amendable to the initial treatment plan and may require a different size or radial force stent (i.e., self-expanding vs. balloon expandable) to obtain the reference vessel diameter which the CMSB system also provides.

### Current venous sizing and stenting challenges

The average iliac venous flow is relatively constant in adults with a narrow range (11% of cardiac output ± 20%). Therefore, the minimum desirable iliac vein caliber to maintain normal venous pressure (<11 mm Hg) can be estimated from the Poiseuille equation and scaling rules. By this method, the ‘optimal’ stent calibers (diameter) for the common and external iliac veins have been estimated to be between 16 mm and 14 mm (Raju et al., 2018). This is not a refined method and does not account for individual variations in wall properties, flow, or collateralization. It is only a single-point snapshot in the non-linear compliance curve. Furthermore, the curve itself will change from the baseline to post-balloon dilatation and stenting, evolving slowly thereafter during the remodeling of the treated vein. An understanding of the comprehensive compliance regimen of the target iliac vein segment is necessary for diagnosis, proper stent sizing, and post-stent surveillance. Currently, no easy, clinically applicable method exists to derive a venous compliance curve over the physiologic pressure range.

Stent under-sizing and oversizing result in adverse clinical outcomes. Poor venous compliance can contribute to stent recoil and significantly affect stent diameter and accurate stent deployment (Saleem et al., 2023). This also sets the stage for restenosis and potential migration as the lesion and recoil squeezes the stent away spontaneously or during post-dilatation (Raju et al., 2014). Improper sizing and delivery of the stent may also result in its excessive protrusion into the iliocaval confluence and “jailing” of the contralateral iliac vein; this may lead to its thrombosis extending further distally in the contralateral limb. Additionally, regions of compression at stent ends or elongation have been associated with restenosis and ipsilateral deep venous thrombosis. This relates to inadequate stent expansion against the poorly compliant diseased venous segments (Raju et al., 2014).

After venous stenting, 25%–29% of patients require one or more reinterventions commonly to correct in-stent-stent restenosis (ISR) of Wallstent that have been used widely in this location (Raju et al., 2009; van Vuuren et al., 2018; Raju et al., 2019). Wall shear-stress abnormalities are associated with this pathology (Jayaraj et al., 2022). The precise cause of ISR is unknown. It occurs more commonly in stents deployed in post-thrombotic limbs (Neglen and Raju, 2004). It is often found at misaligned stent joints where ‘shelving’ is present (Saleem et al., 2022). Compliance information during the development of the lesion can provide further insights into the cause and nature of this lesion and hence the motivation for the current study.

### Potential clinical applications of CMSB system

There are numerous complexities to iliac venous stenting. An ‘ideal’ stent for iliac-caval-femoral application will have unique requirements to meet the complex anatomy, variable pathology, and caliber variations specific to the individual patient.

#### Complex venous anatomy

The iliac veins trace a complex spiral in the pelvis with continuous change in all axes of its three-dimensional orientation. In addition, a series of ‘choke’ bands of adjacent arteries or ligaments cross the vein, reducing its caliber and compliance at the crossover points (Raju et al., 2009). The most prominent choke point is the right common iliac artery crossing the left common iliac vein at the iliac-caval junction, commonly referred to as May-Thurner syndrome. Even in asymptomatic individuals, a variable caliber stenosis ranging from 10% to 75% is present at this crossover point (Kibbe et al., 2004). Additional choke points at the hypogastric cross-over point and behind the inguinal ligament also produce variable stenosis of the iliac-femoral vein segments. The iliac-caval confluence with variable anatomy poses unique challenges, whether unilateral or bilateral iliac vein stenting (Negus et al., 1968; Raju et al., 2014).

#### Variable pathology

Non-malignant iliac vein stenosis is predominantly due to two types of pathology: 1) Non-PT iliac vein lesion (NIVL) caused by traumatic injury of the vein by the pulsations of the closely related crossing artery and 2) PT stenosis resulting from variable resolution of organizing thrombus. NIVL, referred to as iliac vein “compression,” often involves mural and intraluminal fibrosis in addition to external compression. The stenosis (presumably compliance changes as well) is subsegmental (Raju and Neglen, 2006). Isolated PT stenosis of the common femoral vein is quite rare without iliac vein involvement (Neglen et al., 2008). The PT process is associated with pre-existing NIVL in about 10% of limbs. Thrombus resolution lags at the NIVL bands adding to the severity of stenosis. The CMSB system is expected to show different compliance patterns in these variable pathologies. Phantom iliac vein stenosis resulting from imaging artifacts should exhibit normal compliance with CMSB.

#### Intraprocedural use

The CMSB system is expected to function as a valuable tool during iliac vein balloon/stenting procedures, including re-interventional procedures. At present, balloon/stenting of iliac vein stenosis is somewhat of a blind procedure. The lesion is ballooned to *ad hoc* diameters and pressures, and the result is surveyed by venography or intravascular ultrasound. Multiple dilatations are typical to ensure that adequate relief of the stenosis has been obtained (Brodie et al., 2003). The procedure can be simplified with savings of supplies and cost if the compliance profile obtained by the CMSB system is available. The human iliac veins are typically 15 cm long. The CMSB system can delineate areas of high and low compliance. In a minority of cases, the disease is confined to only one of the two segments, in which case balloon dilatation and associated injury of the uninvolved segment can be avoided (Raju et al., 2020). CMSB system can provide useful information on the size and pressure rating of the balloon necessary to correct the stenosis. It can ensure that a post-balloon reading has achieved adequate relief of stenosis. Most iliac vein lesions in patients are relieved by a balloon pressure of 2 ATM or less. Some lesions, particularly PT lesions or recurrent in-stent stenosis, may require up to 16 ATM balloon pressure for adequate relief (Raju et al., 2019). The experimental PT stenosis used in the current study may not fully reflect the more complex and variable pathology of human disease.

#### Individual variations

The stent should be size-matched to approximate the individual-specific normal iliac vein size to decompress the periphery and be flexible to accommodate the complex anatomical 3D curvature but rigid enough at various choke points to resist compression. The stent should also accommodate contralateral iliac flow without restriction and be readily deployable in bilateral applications. Local regions of compression determine the extent of stent recoil (estimated at 20%–30%) and hence, inaccurate delivery and foreshortening of the stent. This will have subsequent restenosis consequences. The requirement to prevent stent recoil from lesion compression is particularly critical at the stent ends, where narrowed ostia can lead to migration as the stent is pressed away from the lesion (Raju et al., 2014).

Accurate sizing of the vein cross-sectional area (CSA) and compliance measurement along the lesion or multiple adjacent lesions is essential. This may indicate the need for a stent with axially variable stiffness or radial force, i.e., more radial force where needed and less when unnecessary. Under-sizing a stent is more harmful than slight oversizing (albeit significant oversizing can risk stent erosion and remodeling). Under-sizing the iliac vein stent is a cause of permanent iatrogenic stenosis that is not easily corrected (Saleem et al., 2023). A CMSB system adequately rigged for human use is expected to provide information on proper stent sizing during post-dilatation reading. The advent of accretive manufacturing offers the future prospect of custom-made stents to suit the individual compliance profile provided by the CMSB system. Technical defects such as shelving at stent joints may be ‘hidden’ on post-stent venography but should be apparent with CMSB system use.

#### Reinterventions

Re-interventional correction is often required with wall stents which are the most used in the iliac veins. The reason for reintervention includes stent compression from restenosis or, more commonly, from in-stent restenosis (Jayaraj et al., 2022). There is considerable literature on in-stent restenosis of arterial stents. Much less is known about in-stent restenosis in the venous system. There is endothelial remodeling, wall thickening, and reduced compliance in response to induced reflux/venous hypertension in the canine iliac veins (Brass et al., 2015). Adventitial and medial fibrosis lined by thrombus are features of venous in-stent restenosis (Raju et al., 2009; Raju et al., 2014; Raju et al., 2019). Intimal hyperplasia, prominent in arterial in-stent restenosis, is absent or minimal in venous lesions. It is unclear if the venous lesions are pathologically like the arterial variety. CMSB system profile in these cases will be of major interest. *In vivo* compliance measurements in the human iliac veins have not been reported. Imaging tools, including IVUS, computed tomography, etc., cannot identify differences in lesion morphology (soft versus hard) or vessel compliance. Hence, there is a significant need for a technology that can accurately assess the lumen profile (CSA), along with axial variations in the compliance of iliac veins, perioperatively and during the procedure. As the main standard of care measurement, IVUS is more accurate than venography and provides a local vessel assessment of geometric complexities, but in addition to the limitation of being unable to measure compliance noted above, IVUS also requires detailed training and image interpretation. In contrast to IVUS assessment, the CMSB system is based on a physical law (Ohm’s Law) that leverages a small/safe voltage measurement made in real-time inside the balloon without the aid of the user. The conductance technology overcomes inaccurate determination of size based on manufacturer pressure/diameter relationships. The CMSB pre-dilatation catheter requires no calibration or training and provides objective metrics without user intervention. Another major limitation of IVUS is the absence of a centering mechanism. This often results in tilting of the catheter tip at the Iliac-IVC confluence yielding an incomplete image of the stenotic lesion. In one study as many as about 20% of IVUS identified lesions could not be completely imaged (Montminy et al., 2019).

### Study limitations

The CMSB system described herein for swine to correct experimental PT stenosis is a useful basic template but will likely require modifications to meet human specifications. As detailed above, the human anatomy and pathology are different from the swine model. Fortunately, the technology is flexible to accommodate various diameters and lengths of interest.

### Summary and conclusions

Our study confirmed that the CMSB technology has several advantages, including Accuracy (Ohm’s law physics basis versus empirical pressure/diameter relationships), time (real-time digital sizing display), efficiency (no significant training required), and potential for future clinical applications (e.g., post dilatation) of related to venous stenting. This study merits the advancement of the technology towards a pilot human study which should improve treatment for numerous patients that require venous stenting.

## Data Availability

The original contributions presented in the study are included in the article/supplementary material, further inquiries can be directed to the corresponding author.
